# Supplementary material for: THz emission from Fe/Pt spintronic emitters with L10-FePt alloyed interface
Source: iScience. 2022 Apr 29;25(5):104319. doi: 10.1016/j.isci.2022.104319 (PMC9114522; doi:10.1016/j.isci.2022.104319)
Supplement: Document S1. Figures S1–S4 [file mmc1.pdf]

iScience, Volume *25*

Supplemental information

THz emission from Fe/Pt spintronic emitters with $L1_0$-FePt alloyed interface

Laura Scheuer, Moritz Ruhwedel, Dimitrios Karfaridis, Isaak G. Vasileiadis, Dominik Sokoluk, Garik Torosyan, George Vourlias, George P. Dimitrakopoulos, Marco Rahm, Burkard Hillebrands, Thomas Kehagias, René Beigang, and Evangelos Th. Papaioannou

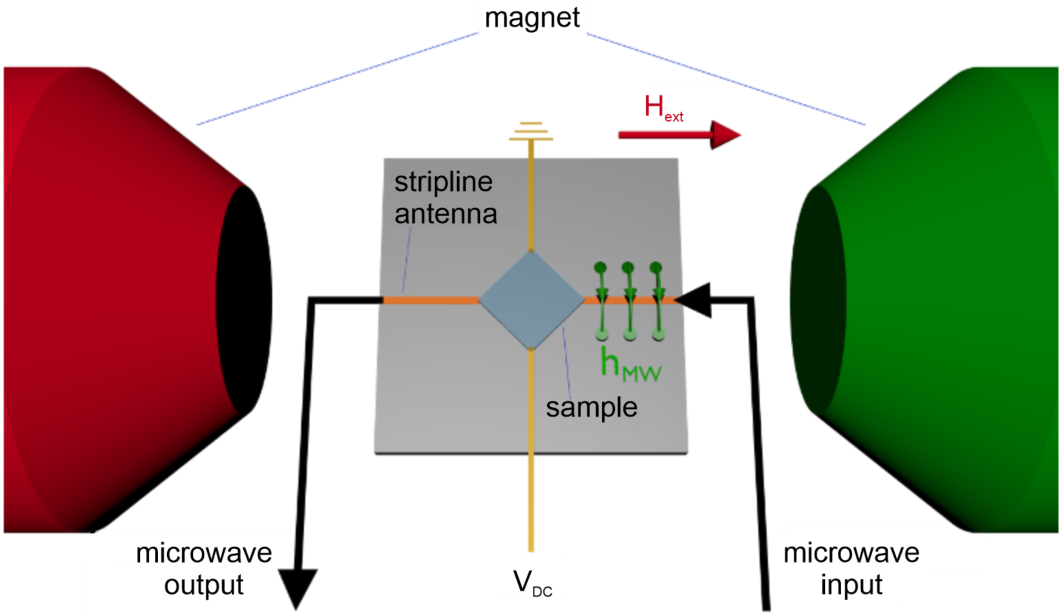

Supplementary FIG. **S1**. Schematic representation of the Vector Network Analyser (VNA)-FMR experimental setup. The magnetization dynamics is induced by a microwave current flowing through the microwave stripline. The stripline with the sample is placed between the poles of an electromagnet such that the line is parallel to the direction of the applied static magnetic field (in-plane FMR configuration). The stripline is connected to a VNA functioning as a microwave signal source to excite magnetization dynamics as well as a signal receiver. Related to Key resource table: FMR evaluation, Star Methods, and to Figure S2.

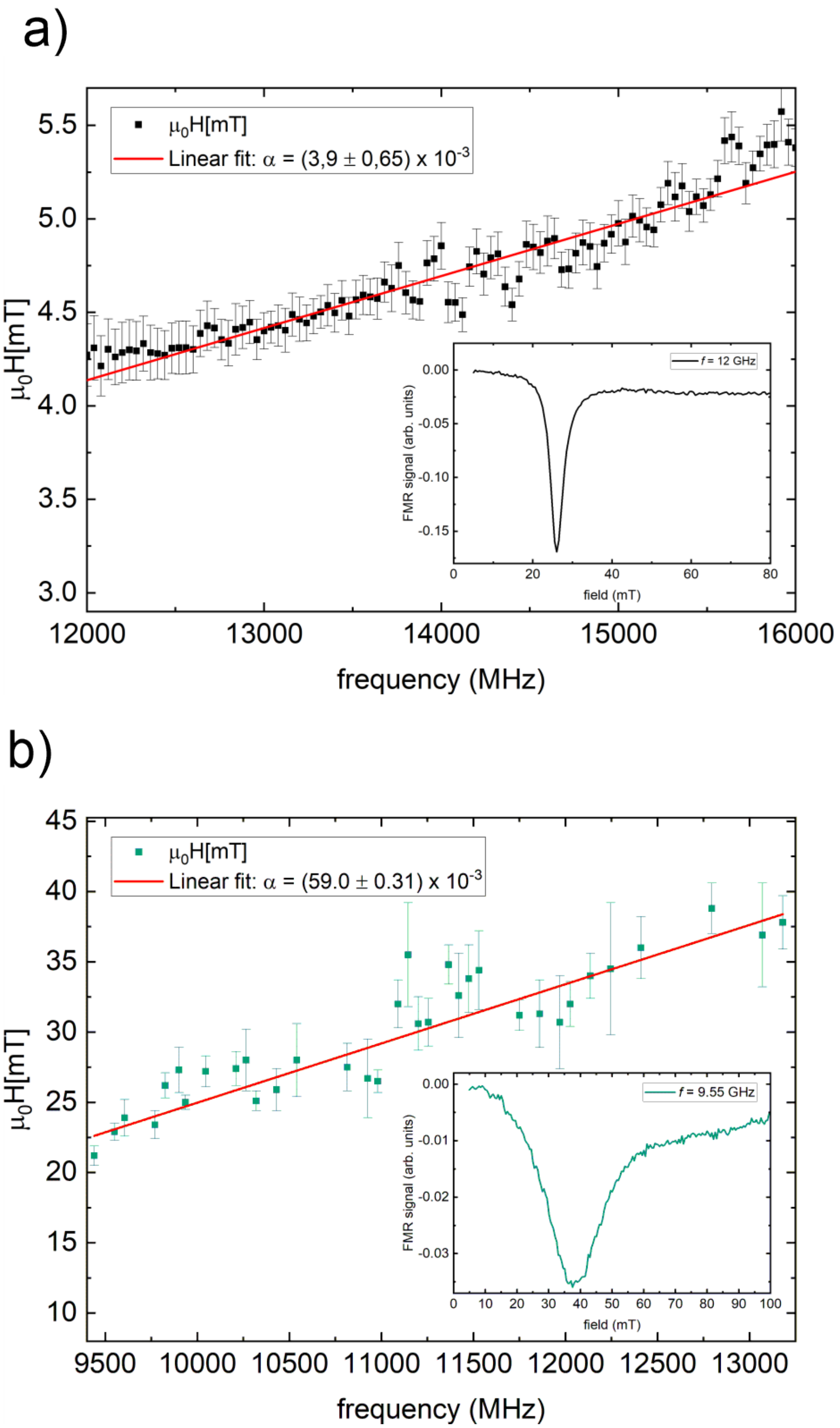

Supplementary FIG. **S2**. Damping calculation and examples of FMR absorption spectra for a) Fe/Pt and b) Fe/L1_0-FePt/Pt samples. From the dependence of the resonance linewidth on the frequency the damping parameter α is determined. The red line is a linear fit according to Eq. 3. Insets show typical FMR absorption spectra for both samples. Related to Figure 5.

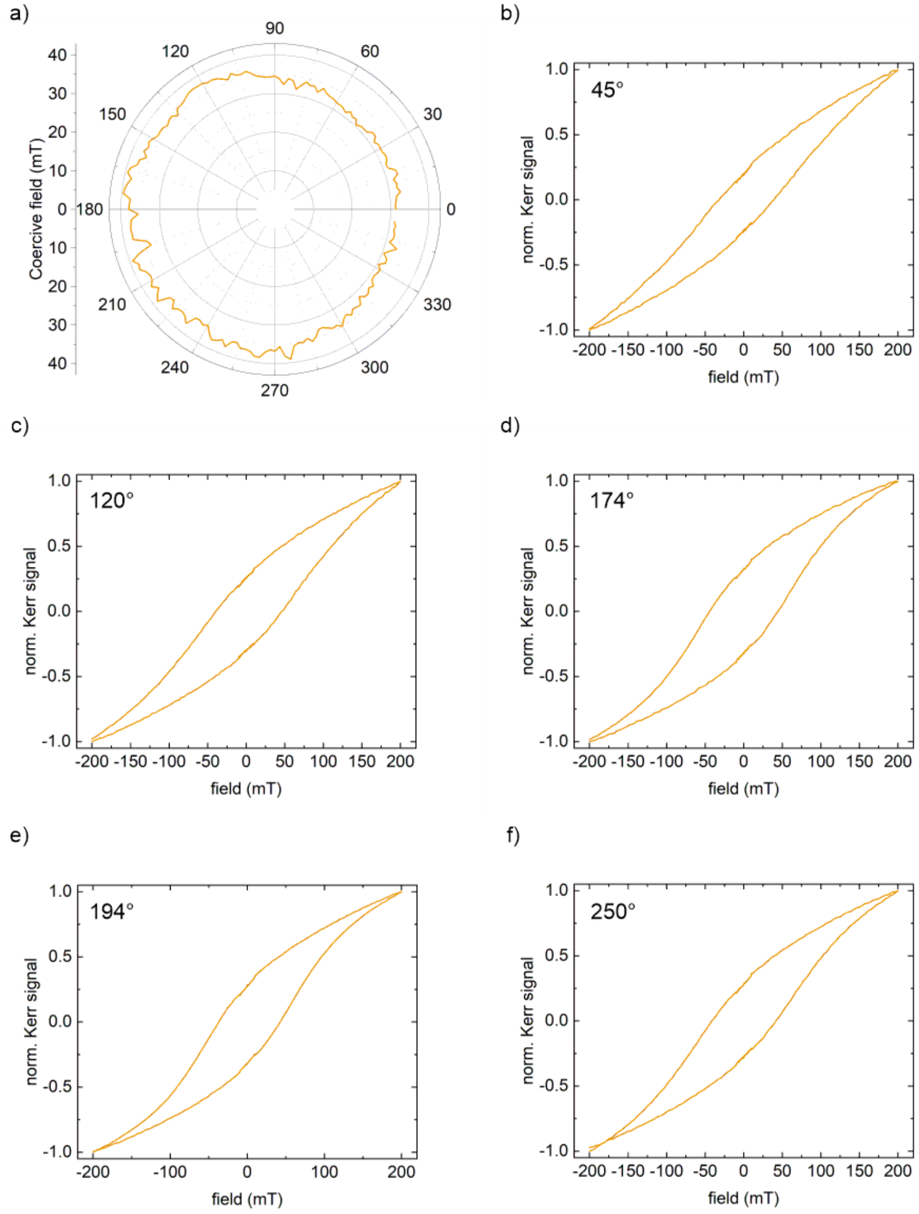

Supplementary FIG. **S3**. Magnetic hysteresis loops measured with in-plane (longitudinal) swept magnetic field (Longitudinal Magnetooptical magnetometry (L-MOKE)) for the sample grown at 600°C. The maximum available applied external magnetic field of the setup of 200 mT is not able to saturate the strongly alloyed sample. Furthermore, by rotating the magnetic field in-plane no change in the magnetization reversal was observed. The high coercive field values remain largely constant as the polar plot in Fig. S3(a) shows. Related to Figure 4 and Figure 5.

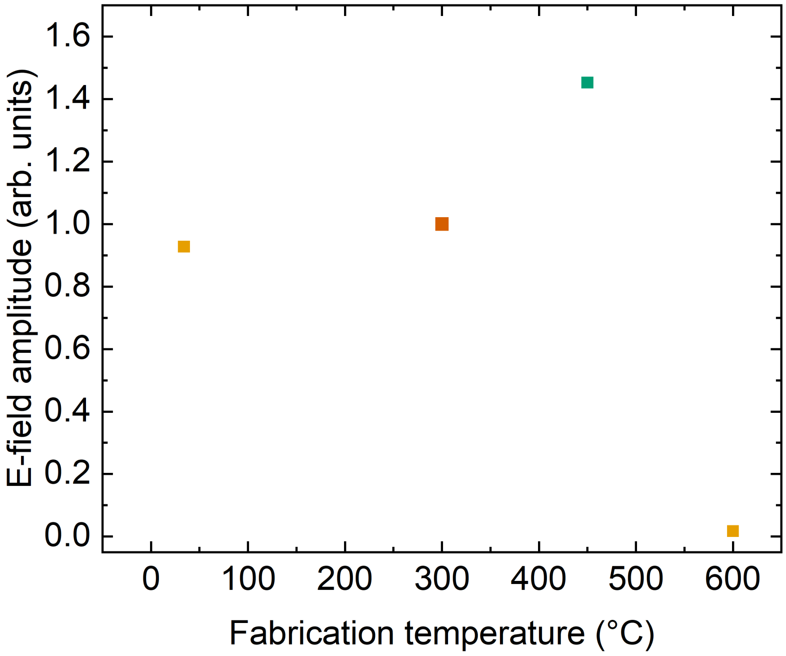

Supplementary FIG. **S4**. E-field amplitude of the THz emission (peak-to-peak value) from a sample series of Fe (12 nm)/ Pt (6 nm) with the Pt layer grown at RT, 300°, 450°, 600°. The Fe layer is grown at 300° for all samples. The data are normalized to the value of Fe (12 nm) / Pt (6 nm) grown at 300° which is found to be the most efficient temperature for THz emission for a bilayer of these thicknesses [Ref.: Nenno et al., Scientific Reports 9, 13348 (2019)]. We observe that the trilayer Fe/L1_0-FePt/Pt is superior to all other Fe/Pt samples grown at different temperatures. Related to Figure 5.
